# The Effect of High-Definition Transcranial Direct Current Stimulation of the Right Inferior Frontal Gyrus on Empathy in Healthy Individuals

**DOI:** 10.3389/fnhum.2018.00446

**Published:** 2018-11-12

**Authors:** Xiaoling Wu, Feifei Xu, Xingui Chen, Lu Wang, Wanling Huang, Ke Wan, Gong-Jun Ji, Guixian Xiao, Sheng Xu, Fengqiong Yu, Chunyan Zhu, Chunhua Xi, Kai Wang

**Affiliations:** ^1^Department of Medical Psychology, Chaohu Clinical Medical College, Anhui Medical University, Hefei, China; ^2^Anhui Province Key Laboratory of Cognition and Neuropsychiatric Disorders, Hefei, China; ^3^Collaborative Innovation Center for Neuropsychiatric Disorders and Mental Health, Anhui, China; ^4^Department of Neurology, The First Affiliated Hospital of Anhui Medical University, Hefei, China; ^5^Department of Neurology, The Third Affiliated Hospital of Anhui Medical University, Hefei, China

**Keywords:** cognitive empathy, emotional empathy, high-definition transcranial direct current stimulation, inferior frontal gyrus, Multifaceted Empathy Test

## Abstract

Empathy, including cognitive and emotional empathy, refers to the ability to infer the mental states of others and to the capacity to share emotions. The neural mechanisms involved in empathy are complex and not yet fully understood, and previous studies have shown that both cognitive and emotional empathy are closely associated with the inferior frontal gyrus (IFG). In this study, we examined whether empathy can be modulated by high-definition transcranial direct current stimulation (HD-tDCS) of the right IFG. Twenty-three healthy participants took part in all three experimental conditions (i.e., anodal, cathodal and sham stimulation) in a randomized order. Participants then completed the Chinese version of the Multifaceted Empathy Test (MET), which assesses both cognitive and emotional empathy. The results show that scores obtained for cognitive empathy following cathodal stimulation are significantly lower than those obtained following sham stimulation. In addition, scores obtained for cognitive empathy following anodal stimulation are higher than those obtained following sham stimulation, though the difference is only marginally significant. However, the results fail to show whether the stimulation of the right IFG via HD-tDCS plays a role in emotional empathy. Our results suggest that the right IFG plays a key role in cognitive empathy and indicate that HD-tDCS can regulate cognitive empathy by inducing excitability changes in the right IFG.

## Introduction

As a sociocognitive ability, empathy plays a very important role in our interpersonal interactions (Decety and Cowell, 2014). Empathy involves both cognitive and emotional components that correspond to two abilities, the first of which is the ability to infer the mental states of another (i.e., “I understand what you feel”) and is described as cognitive empathy (Decety and Lamm, 2006; Harvey et al., 2013). The second is the ability to respond to the observed emotions of others or to share a “fellow feeling” (i.e., “I feel what you feel”) and is known as emotional empathy, which includes emotional recognition, emotional contagion, and the sharing of pain (Shamay-Tsoory et al., 2009; Shamay-Tsoory, 2011). Current evolutionary evidence supports the theory that emotional empathy develops earlier than cognitive empathy, the latter of which involves higher levels of cognitive functioning (Shamay-Tsoory et al., 2009; Shamay-Tsoory, 2011). There is still controversy regarding the role of empathy in our daily lives and such debate mainly focuses on the relationship between empathy and morality. Bloom believed that empathy can produce moral prejudice, which can cause individuals to experience personal biases toward those whom they are close to or familiar with when making moral decisions (Bloom, 2016, 2017). However, some researchers have opposed this view by arguing that empathy is a moral force that can motivate engagement in prosocial behavior (Zaki, 2016, 2017). Zaki (2016), for example, argued that prejudice resulting from processes of empathy described in Bloom is not the cause of empathy itself but rather reflects the motivations of the individual. Despite this controversy, most current studies have supported the important role of empathy in our lives in enabling us to accurately recognize the emotions and behaviors of others and to respond appropriately (Fan et al., 2011). Empathic impairment not only seriously affects the daily lives of people but also leads to many serious social problems (Zaki, 2016). Therefore, the study of empathic impairment is crucial to the study of empathy.

The neural mechanisms involved in empathy are complex and not yet fully understood. In recent years brain lesion and imaging studies have been widely used to explore the neural mechanisms that underlie empathy. Research has shown that cognitive empathy is generally supported by the activation of the medial prefrontal cortex (MPFC), right temporoparietal junction (TPJ), inferior frontal gyrus (IFG), supplementary motor area and anterior midcingulate cortex (aMCC; Völlm et al., 2006; Schulte-Rüther et al., 2007, 2008; Hooker et al., 2008, 2010; Massey et al., 2017) while emotional empathy involves the activation of brain regions such as the IFC, anterior insula (INS), anterior cingulate cortex (ACC) and superior temporal sulci (STS; Schulte-Rüther et al., 2008; Shamay-Tsoory et al., 2009; Shamay-Tsoory, 2011; Leigh et al., 2013; Oishi et al., 2015). Shamay-Tsoory et al. (2009) found that patients who have suffered damage to the ventral MPFC experience low levels of cognitive empathy and that those who have sustained damage to the IFC exhibit low levels of emotional empathy. Moreover, magnetic resonance imaging (MRI) studies of the neural mechanisms underlying empathy are extensive. Massey et al. (2017) found that the cortical thickness of the mPFC, right IFG, aMCC, INS and left TPJ in healthy subjects is significantly associated with cognitive empathy. Pfeifer et al. (2008) found the significant activation of the right IFG, right INS and right amygdala in children during engagement in empathic behavior and showed that the activation of the right IFG is significantly associated with the Interpersonal Reactivity Index (IRI), a self-reported empathy questionnaire. Schulte-Rüther et al. (2007) also found that the mPFC, bilateral IFG and STS are clearly activated during an empathic interpersonal face-to-face interaction task and that the activation of the right IFG and left STS is significantly correlated with scores obtained from participants using two self-reported emotional empathy scales. These studies show that the IFG plays a highly significant role in the brain mechanisms that underlie empathy.

As far as we know, measurements of empathy used in past studies have mainly included self-reported scale and behavioral task measurements. The most widely used self-reported scale is the IRI scale (Davis, 1980), which measures both cognitive and emotional empathy. However, during engagement in empathic behavioral tasks, most tasks cannot measure cognitive and emotional empathy simultaneously, as they typically measured a single form of empathy like cognitive empathy (Baron-Cohen et al., 2001; Smith et al., 2015; Mai et al., 2016; Massey et al., 2017; Oliver et al., 2018) and emotional empathy (Derntl et al., 2012; Smith et al., 2014). To address this limitation, Dziobek et al. (2008) developed a new measure of empathy, the Multifaceted Empathy Test (MET), which is designed to measure both cognitive and emotional empathy. The MET is a well-established task that has been widely used in the study of healthy subjects (Hysek et al., 2014; Ze et al., 2014; Kuypers et al., 2017) and of those with various psychiatric disorders, including patients diagnosed with schizophrenia (Lehmann et al., 2014), depressive disorder (Wingenfeld et al., 2016), autism spectrum disorder (Dziobek et al., 2008; Mazza et al., 2014), and borderline personality disorder (Harari et al., 2010; Dziobek et al., 2011; Wingenfeld et al., 2014). The present study uses the Chinese version of the Multifaceted Empathy Test (MET-C) revised by Zhu et al. (2018), which is highly internally reliable and valid. The MET-C is stronger in ecological validity than self-report questionnaires that measure empathy, as the test measures several photorealistic stimuli (Dziobek et al., 2008). Therefore, the MET-C serves as a better behavioral paradigm for measuring empathy.

Studies have shown that empathic impairment affects people’s social interactions, and studies of psychiatric patients have found that empathic impairment is a core deficit observed in psychiatric disorders that directly leads to the severe impairment of their social functions (Dapretto et al., 2006; Derntl et al., 2009; Smith et al., 2015). Thus, the present study considered neuromodulatory technologies are used to enhance empathic abilities. Transcranial direct current stimulation (tDCS) is a noninvasive neurostimulation technique that involves applying a weak direct current (usually 1–2 mA) through electrodes placed on the scalp that can alter the activity and excitability of cortical neurons, thereby inducing changes in neural functioning. Stimulation polarity determines the direction of cortical excitability changes, and anodal stimulation can generally increase the excitability of the cortex while cathodal stimulation has the opposite effect. Conventional tDCS involves the placement of two large sponge electrodes (25–35 cm^2^; one anode and one cathode electrode) onto two different areas of the scalp such that the current flows from the anode to the cathode (Nitsche and Paulus, 2001; Nitsche et al., 2003; Hogeveen et al., 2016; Godinho et al., 2017). However, studies have shown that conventional tDCS currents are relatively diffuse and cannot focus currents on the target area of interest due to their weak levels of spatial focality (Antal et al., 2014; Meinzer et al., 2014). However, more recent developments in high-definition tDCS (HD-tDCS) have sought to address this shortcoming (Datta et al., 2008, 2009). HD-tDCS uses arrays of five small circular electrodes (1 cm diameter) rather than traditional large sponge electrodes and applies a 4 × 1 ring electrode configuration with a central electrode positioned over the target brain region and with four return electrodes (each receiving 25% of the return current) positioned around it, thus allowing currents to enhance brain targeting in the area surrounded by return electrodes (Datta et al., 2009; Minhas et al., 2010; Hogeveen et al., 2016). In short, HD-tDCS ensures higher levels of spatial focality than conventional tDCS, which can offer a better understanding of the causal relationship between changes in brain excitability and subsequent changes in behavioral or cognitive ability (Hogeveen et al., 2016).

In recent years, tDCS has been widely used in research studies to investigate the social cognitive abilities of healthy subjects (Nitsche et al., 2012; Willis et al., 2015; Mai et al., 2016; Adenzato et al., 2017; Martin et al., 2017) and of psychiatric patients (Brennan et al., 2017; Philip et al., 2017). Studies have shown that anodal stimulation can typically enhance certain social cognitive abilities while cathodal stimulation has the opposite effect. Until now only a few research studies have employed tDCS to investigate empathy in healthy subjects (Wang et al., 2014; Rêgo et al., 2015; Mai et al., 2016; Coll et al., 2017; Nobusako et al., 2017). Mai et al. (2016) found that the cathodal tDCS stimulation of the right TPJ inhibits the capacity for cognitive empathy. Furthermore, Nobusako et al. (2017) found that the anodal tDCS of the right IFC enhances capacities for perspective-taking (PT), which is used to evaluate cognitive empathy. However, no studies have applied tDCS to examine emotional empathy. In short, the above findings suggest that tDCS-induced cortical excitability can modify cognitive functioning.

From the above studies we know that the IFG (particularly the right IFG) plays an important role in empathy (Lawrence et al., 2006; Schulte-Rüther et al., 2007, 2008; Pfeifer et al., 2008; Massey et al., 2017; Nobusako et al., 2017). Thus, we can explore the relationship between changes in IFG activity and empathic ability through HD-tDCS, and changes in empathy as a result of HD-tDCS may be further assessed by using the MET-C. Accordingly, our hypothesis suggests that the anodal HD-tDCS stimulation of the right IFG may enhance cognitive and emotional empathy while the cathodal stimulation may have the opposite effect.

## Materials and Methods

### Participants

Twenty-four healthy right-handed adults (mean age 24.39 ± 3.47 years; 16.65 ± 1.66 years of education; 17 females) participated in the study. One of the male participants withdrew from the study after the first stimulation. As such, complete and reliable data were obtained for 23 participants. All participants had normal vision and none were colorblind. None of the participants had a history of neurological or psychiatric illness, head injury, alcohol dependance, or drug dependance. Participants took no psychoactive drugs, experienced no illnesses or major life events that caused significant changes in their mood, and did not smoke or drink for the duration of the experiment. The study was approved by the Medical Ethics Committee of Anhui Medical University, Hefei, China. Each participant provided their written informed consent prior to the study.

### Measures

#### Neuropsychological Assessment

The basic cognitive functioning and emotional conditions of the participants were assessed by administering standardized neuropsychological tests. The IRI was used to measure the cognitive and emotional empathic traits of the participants such that cognitive empathy was assessed using PT and Fantasy (F) subscales while emotional empathy was measured using subscales for empathic concern (EC) and personal distress (PD; Davis, 1980). The Hamilton Anxiety Scale (HAMA) and Hamilton Depression Scale (HAMD) were used to evaluate potential anxiety and depressive symptoms experienced by the participants. Finally, overall cognitive functioning was measured through a Montreal Cognitive Assessment (MoCA) test.

#### Behavioral Measurement

**Figure 1:**
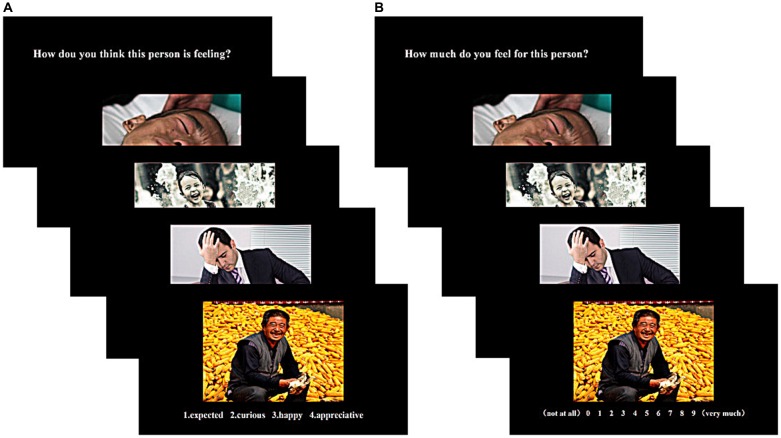
Tasks for measuring cognitive empathy **(A)** and emotional empathy **(B)**. Stimuli were presented in blocks of 10. Each block was introduced with a question indicating the block type.

The MET-C was administered to assess the two components of empathy, cognitive and emotional empathy. The task involved the presentation of 40 emotional pictures of adults and children of both genders. The images included 20 positive and 20 negative pictures (valence) and most portrayed a particular social context (see Figure 1). Measurements of both cognitive and emotional empathy were taken in four blocks, producing a total of eight blocks. Each block involved 10 trials, resulting in a total of 80 trials. Participants were asked to answer two questions. For the cognitive empathy assessment, participants were asked to judge the emotional states of the individuals shown in the pictures based on the given social context (social clues) and the facial expressions of the individuals (facial clues) and to select the most appropriate answer from four emotional state descriptors. Scores for correctly answered questions ranged from 0 to 40 and accuracy and response times (RTs) were recorded for each trial that assessed cognitive empathy. For the emotional empathy assessment, participants were asked to evaluate how much they experienced the emotions of the individuals shown in the pictures on a scale of 0–9 (0 = not at all, 9 = very much). The average rating was calculated to produce a score for emotional empathy. Prior to completing the formal experiment, all participants received brief training to ensure that they had a thorough understanding of the task requirements.

### HD-tDCS Stimulation

**Figure 2:**
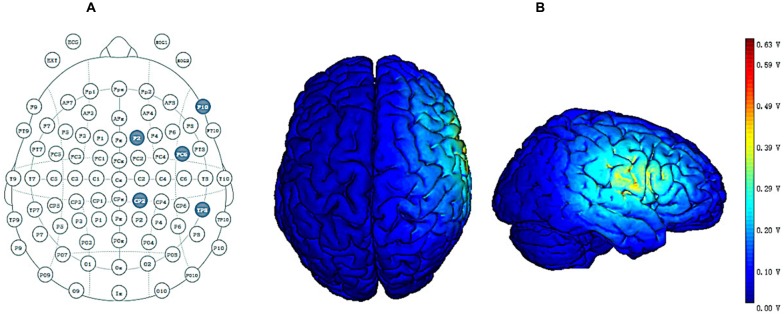
**(A)** High-definition transcranial direct current stimulation (HD-tDCS) electrode position. FC6 represents the central electrode, and F2, F10, CP2 and TP8 represent return electrodes. **(B)** Stimulated distribution of the electric field in cortical gray matter with the selected montage.

HD-tDCS was administered through a battery-driven constant-current stimulator (Neuroelectrics, Barcelona, Spain). Based on previous studies and the International 10/20 EEG System, as the target region (anode or cathode electrode) for stimulation we selected FC6 (Hogeveen et al., 2015, 2016; Nobusako et al., 2017) and return electrodes were placed in four locations around the central electrode corresponding to F10, CP2, TP8 and F2 (Figure 2A; Hogeveen et al., 2016). The distance between each return electrode and central electrode was measured as ~6 cm. Under the active HD-tDCS condition, a relatively weak current (1.5 mA) was delivered for 20 min. For sham stimulation, a 1.5 mA current stimulus was delivered and lasted only 30 s consistent with previous research (Civai et al., 2015; Mai et al., 2016; Tang et al., 2017). For all three stimulation conditions, the stimulation commenced with the delivery of a current that slowly increased from 0 mA to 1.5 mA (ramp-up duration of 15 s) and that slowly dropped from 1.5 mA to 0 mA at the end of the stimulation (ramp-down duration of 15 s; Cerruti and Schlaug, 2008; Holland et al., 2011). The slow rise and fall in current allowed the participants to adapt gradually to the stimulation, thereby avoiding experiencing a tingling sensation from the current (Zito et al., 2015).

Figure 2B shows the distribution of the electrical field across cortical gray matter for the selected montage. The distribution of the electrical field produced by HD-tDCS is concentrated in the right prefrontal region, and the maximal intensity of 0.63 V/m occurs around the central electrode while the current flow is largely limited to the area defined by return electrodes. These findings are consistent with previous HD-tDCS studies showing that HD-tDCS offers enhanced levels of spatial focality.

### Experimental Design

**Figure 3:**
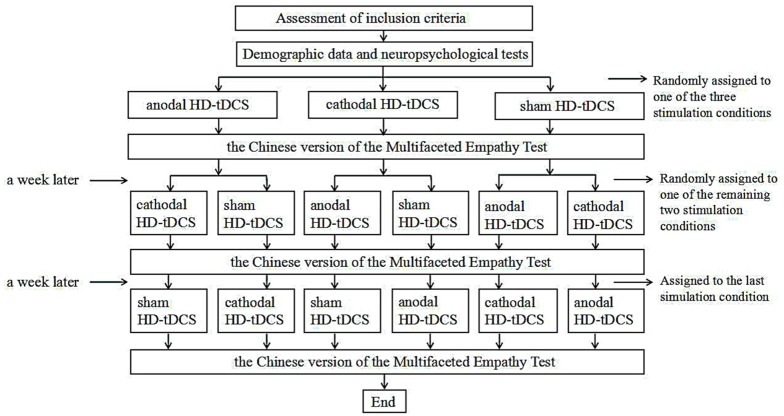
Experimental design of the single-blind, within-subject and sham control trial.

We employed a single-blind, sham-controlled, within-subject study design. Each participant underwent all three experimental conditions (i.e., anodal, cathodal, and sham stimulation) in a randomized order. Prior to the first stimulation, all participants provided basic demographic information and underwent neuropsychological tests. They then randomly received HD-tDCS stimulation (anodal, cathodal and sham stimulation). During stimulation, participants were asked to sit alone in a quiet room to prevent the external environment from affecting the experimental results. After the stimulation, the MET-C test was immediately performed. During the test, every participant maintained a good sitting posture to ensure that his or her finger could easily touch the computer keyboard to select a choice. All participants completed a total of three stimulation periods which were held at least 7 days apart to discourage practice and memory effects. Each experiment lasted ~40 min including 10 min of preparation, 20 min of stimulation, and 10 min designated for the behavioral task. Figure 3 illustrates this experimental design.

### Data Analysis

A data analysis was performed using SPSS 18.0 (IBM, Armonk, NY, USA). Data on behavioral measures of accuracy, RTs for the cognitive empathy task and average ratings for the emotional empathy task were analyzed through a repeated-measures analysis of variance (ANOVA) with two independent within-subject factors (stimulation conditions, 3; valence, 2). When sphericity violation occurred, Greenhouse-Geisser corrections were performed. Further *post hoc* analyses were carried out using Fisher’s Least Significant Difference test where appropriate. A Pearson correlation coefficient (*r*) was used to assess the correlation between the personality measures and HD-tDCS effects on cognitive and emotional empathy. For all of the statistical tests, the alpha level was defined as *p* < 0.05.

## Results

### Demographic Data and Neuropsychological Tests

This section presents a summary of the participants’ demographic information and the results of neuropsychological assessments (age 24.39 ± 3.55, years of education 16.64 ± 1.73, HAMA 3.48 ± 2.11, HAMD 2.65 ± 2.60, MoCA 28.17 ± 1.53, IRI total score 51.13 ± 8.85, PT 11.13 ± 3.09, F 15.48 ± 2.91, EC 16.52 ± 3.87, PD 8.00 ± 3.71). It took all participants ~30 min to complete the assessment.

### Behavioral Results

**Figure 4:**
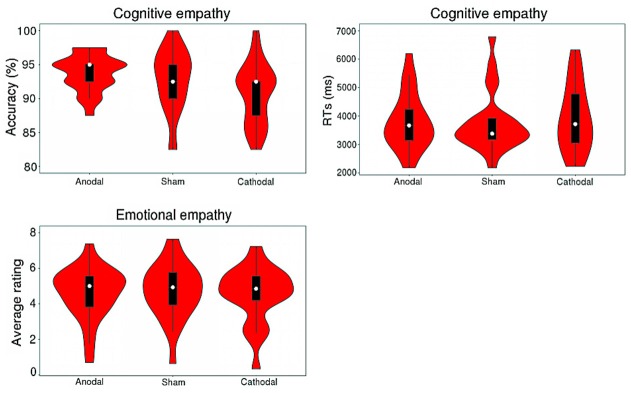
Distribution of the accuracy and response times (RTs) of cognitive empathy and the average rating for emotional empathy for all participants of three stimulations.

Table 1 shows descriptive statistics for cognitive and emotional empathy with two valences for participants who completed all three stimulations. A violin plot (see Figure 4) shows the data distribution and probability density. It combines characteristics of the box plots and histograms due illustrate the distribution of the data. Figure 4 shows the distribution of accuracy levels and RTs for cognitive empathy and average ratings for emotional empathy of all of the participants for all three stimulation conditions.

**Table 1:** Means, standard deviations (SDs) and 95% confidence intervals of Chinese version of the Multifaceted Empathy Test (MET-C) scores for the three stimulation conditions.

Stimulation conditions	Empathy (MET-C)	Valence	Mean	SD	95% Confidence interval	
					Lower	Upper
Anodal	Cognitive empathy			
(*n* = 23)	Accuracy (%)	Positive	94.13	5.15	91.91	96.36
		Negative	93.48	4.63	91.48	95.48
		Total	93.80	2.70	92.63	94.97
	RTs (ms)	Positive	3,415	727	3,101	3,729
		Negative	4,193	1,329	3,618	4,768
		Total	3,804	999	3,372	4,236
	Emotional empathy	Positive	4.62	1.63	3.91	5.33
		Negative	4.54	1.68	3.81	5.27
		Total	4.58	1.49	3.94	5.23
Sham	Cognitive empathy			
(*n* = 23)	Accuracy (%)	Positive	93.04	6.17	90.38	95.71
		Negative	92.17	6.00	89.58	94.77
		Total	92.61	4.62	90.61	94.60
	RTs (ms)	Positive	3,472	960	3,057	3,886
		Negative	4,040	1,190	3,525	4,555
		Total	3,756	1,061	3,297	4,214
	Emotional empathy	Positive	4.81	1.71	4.07	5.55
		Negative	4.61	1.77	3.84	5.37
		Total	4.71	1.57	4.03	5.39
Cathodal	Cognitive empathy				
(*n* = 23)	Accuracy (%)	Positive	92.17	6.54	89.35	95.00
		Negative	89.35	5.70	86.88	91.81
		Total	90.76	4.49	88.82	92.70
	RTs (ms)	Positive	3,550	1,015	3,111	3,989
		Negative	4,273	1,388	3,673	4,873
		Total	3,912	1,181	3,401	4,422
	Emotional empathy	Positive	4.65	1.54	3.99	5.32
		Negative	4.40	1.76	3.64	5.16
		Total	4.53	1.58	3.85	5.21

### Cognitive Empathy

#### Accuracy

**Figure 5:**
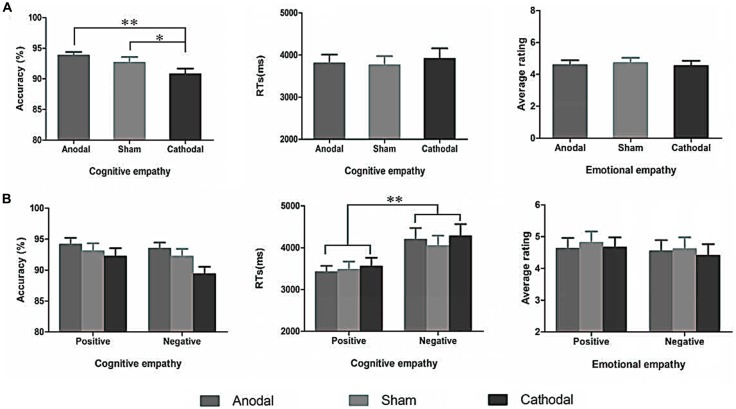
The accuracy and RTs of cognitive empathy, and average ratings of emotional empathy derived from the three stimulation conditions **(A)** and two valences **(B)**. Error bars indicate SEM (standard error of the mean) values, *p < 0.05, **p < 0.01.

A repeated-measures ANOVA shows a reliable main effect of the stimulation conditions (anodal vs. sham vs. cathodal; *F*_(2,68)_ = 8.779, *p* = 0.001, *η*^2^ = 0.285) for accuracy in cognitive empathy. *Post hoc* analyses further reveal a significant difference between the anodal and cathodal stimulation conditions (*p* < 0.001) and between the cathodal and sham stimulation conditions (*p* = 0.038) and show a marginally significant difference between the anodal and sham stimulation conditions (*p* = 0.077; Table 2, Figure 5). Relative to the sham stimulation, the cathodal stimulation generated a lower score for cognitive empathy while the anodal stimulation generated a higher score (Table 1), indicating that the active HD-tDCS stimulation of the right IFC can regulate the accuracy of cognitive empathy. However, the main effect of valence and the interaction effect of stimulation condition × valence were not found to affect accuracy in cognitive empathy.

**Table 2:** Repeated-measures ANOVA on the accuracy and response times (RTs) of cognitive empathy and average ratings of emotional empathy derived from different stimulation conditions and valences.

Factor	*F*	*P*	*η*^2^	95% Confidence interval for difference	
				Lower	Upper
Cognitive empathy					
Accuracy (%)			
Stimulation condition (Anodal vs. Sham vs. Cathodal)	8.779**	0.001	0.285		
Anodal vs. Cathodal		<0.001		1.587	4.500
Anodal vs. Sham		0.077		−0.143	2.534
Cathodal vs. Sham		0.038		0.116	3.579
Valence (positive vs. negative)	1.256	0.274	0.054		
Stimulation condition × valence	0.784	0.463	0.034		
RTs (ms)					
Stimulation condition (Anodal vs. Sham vs. Cathodal)	0.429	0.654	0.019		
Valence (positive vs. negative)	44.752**	<0.001	0.670	−903.528	−475.892
Stimulation condition × valence	1.480	0.239	0.063		
Emotional empathy (max. 9)					
Stimulation condition (Anodal vs. Sham vs. Cathodal)	0.858	0.431	0.038		
Valence (positive vs. negative)	0.504	0.485	0.022		
Stimulation condition × valence	0.362	0.698	0.016		

**p < 0.01.

#### RTs

A repeated-measures ANOVA reveals a reliable main effect of valence (positive vs. negative; *F*_(1,68)_ = 44.752, *p* < 0.001, *η*^2^ = 0.670, Greenhouse-Geisser corrected) for RTs in cognitive empathy (Table 2, Figure 5). Relative to the positive valence, we find slower RTs for stimuli with a negative valence (Table 1). However, the main effect of stimulation conditions and the interaction effect of stimulation conditions × valence were not found for RTs in terms of cognitive empathy, showing that the active HD-tDCS stimulation of the right IFC had no significant effect on RTs in terms of cognitive empathy.

### Emotional Empathy

The results of the repeated-measures ANOVA do not reveal the main effects of stimulation conditions and valence or interaction effects of stimulation conditions × valence on emotional empathy, indicating that the active tDCS stimulation of the right IFC might have no significant modulatory effect on emotional empathy (Table 2, Figure 5).

### Correlation Analyses

**Figure 6:**
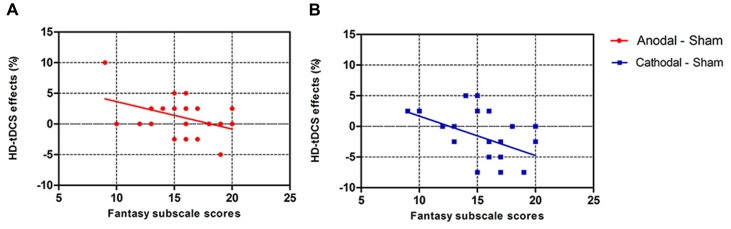
Scatter diagram showing correlations between scores of the Fantasy subscale and HD-tDCS effects for accuracy in cognitive empathy (**A**, anodal *r* = −0.420, *p* = 0.046; **B**, cathodal *r* = −0.468, *p* = 0.024). Note that equal HD-tDCS effects of participants sometimes might be covered by only one data point.

We also analyzed the correlation between the personality measures and HD-tDCS effects (Table 3). Pearson correlation analyses (two-tailed) show that Fantasy subscale scores present a significantly negative correlation with anodal (*r* = −0.420, *p* = 0.046, Figure 6A) and cathodal (*r* = −0.468, *p* = 0.024, Figure 6B) HD-tDCS effects in terms of accuracy in cognitive empathy but that the other personality measures are not significantly associated with HD-tDCS effects whether in terms of cognitive or emotional empathy. The results show lower Fantasy subscale scores and higher anodal and cathodal HD-tDCS effects of accuracy on cognitive empathy, showing that the Fantasy subscale score may play a predictive role in HD-tDCS effects on cognitive empathy.

**Table 3:** Correlation between the personality measures and high-definition transcranial direct current stimulation (HD-tDCS) effects on cognitive and emotional empathy.

Pearson correlation	IRI	PT	*F*	EC	PD	MoCA	HAMA	HAMD
Cognitive empathy								
Accuracy (%)							
Anodal-Sham	−0.097	0.078	−0.420*	0.173	−0.149	−0.214	−0.266	−0.383
Cathodal-Sham	−0.204	0.103	−0.468*	−0.045	−0.161	−0.112	−0.227	−0.261
RTs (ms)								
Anodal-Sham	−0.121	−0.361	0.261	−0.304	0.126	−0.027	−0.237	−0.027
Cathodal-Sham	0.017	−0.067	0.216	−0.200	0.136	−0.187	0.146	0.269
Emotional empathy								
Anodal-Sham	0.052	0.052	0.184	−0.130	0.073	0.042	0.141	−0.246
Cathodal-Sham	0.017	0.400	−0.111	−0.062	−0.141	−0.195	0.290	−0.025

*p < 0.05.

## Discussion

The purpose of this study was to assess the impact of HD-tDCS on cognitive and emotional empathy in healthy participants. HD-tDCS targeting the right IFC was shown to regulate accuracy but with no effect on RTs for cognitive empathy. The accuracy of cognitive empathy for participants of the anodal stimulation test was higher than those of the sham stimulation, though the difference is only marginally significant. The accuracy of cognitive empathy for participants of the cathodal stimulation is significantly lower than that of participants of the sham stimulation. However, the active HD-tDCS of the right IFC had no effect on emotional empathy.

Brain injury and neuroimaging studies of healthy subjects support the important role of the IFG in empathy (Shamay-Tsoory et al., 2003, 2009; Lawrence et al., 2006; Schulte-Rüther et al., 2007, 2008; Pfeifer et al., 2008; Massey et al., 2017). In addition, functional MRI studies of patients with empathic injuries confirm the important role of the IFG in empathy (Dapretto et al., 2006; Smith et al., 2015). Smith et al. (2015) utilized an emotional PT task that assessed cognitive empathy to study the neural mechanisms of empathy in schizophrenia and found that compared to healthy subjects, bilateral IFG activation in the patient group was significantly reduced during the performance of cognitive empathic tasks. Relative to typically developing children, Dapretto et al. (2006) found that the bilateral IFG is not activated in children with autism. The role of the IFG in empathy is also supported by the human mirror neuron system (hMNS). The hMNS is thought to serve as a neural mechanism for understanding others’ intentions, thoughts, actions, and emotions (Fogassi et al., 2005). In addition, the involvement of the hMNS can allow the brain to activate the characterization of observed emotions, thus allowing us to feel the same emotions that we observe in others (Wicker et al., 2003; Jackson et al., 2005). As a core component of the hMNS, the IFG is closely associated with human cognitive and emotional empathy.

Our results indicate that the HD-tDCS of the right IFG has a modulatory effect on the accuracy of cognitive empathy but has no significant effect on RTs. The findings show that the cognitive empathy accuracy of participants receiving cathodal stimulation is significantly lower than that of participants receiving sham stimulation consistent with previous studies employing tDCS (Mai et al., 2016; Coll et al., 2017). Furthermore, cognitive empathy accuracy following anodal stimulation is higher than it is after sham stimulation, though the difference is only marginally significant. This may be the case because MET-C cognitive empathy tasks are easy for healthy subjects to complete. As such, it is difficult to elicit a substantial improvement from HD-tDCS stimulation. In spite of this, the result also implies a potential improvement resulting from anodal stimulation. In short, our study is consistent with previous neuroimaging and brain injury studies (Shamay-Tsoory et al., 2003, 2009; Lawrence et al., 2006; Schulte-Rüther et al., 2007, 2008; Pfeifer et al., 2008; Smith et al., 2015; Massey et al., 2017) and confirms the important role of the IFG in cognitive empathy and the effective role of HD-tDCS in regulating social cognitive functioning. Notably, our results also show that participants responded to negative emotional pictures longer than positive emotional pictures during cognitive empathic tasks consistent with previous studies (Leppänen and Hietanen, 2004; Song et al., 2017; Aldunate et al., 2018). Earlier findings have shown that during emotional recognition, happiness is recognized faster than sadness (Crews and Harrison, 1994; Leppänen and Hietanen, 2004), anger (Hugdahl et al., 1993) and disgust (Stalans and Wedding, 1985). The advantage inherent in responding to positive emotions may result from low levels of physical difference, rendering happy emotions visually more unique and thus easier to identify than others (Leppänen and Hietanen, 2004).

With respect to emotional empathy, the findings are in conflict with the hypotheses of this study, which failed to show that different types of stimulation like HD-tDCS have a significant modulatory effect on emotional empathy. This result may be attributed to the following. First, our task measured emotional empathy in terms of the intensity of emotional mirroring, which reflects only one component of emotional empathy (Oliver et al., 2015), thereby disregarding other measurement components such as EC. Thus, emotional empathy as measured using the MET-C cannot reflect emotional empathy in its entirety. Second, in comparison to cognitive empathy, the measurement of emotional empathy is more subjective. As such, both the external environment and bodily states of the participants had a substantial influence on their responses during the task (e.g., emotional states on the day of the experiment), and due to the small number of participants tested, random error effects were difficult to control, potentially affecting the results of the study. Furthermore, a rating scale of 0–9 was used for the emotional empathy task, which may not have been sensitive enough to evaluate the effects of HD-tDCS on emotional empathy. Finally, our results may simply show that the active stimulation of the right IFG by HD-tDCS has no or little effect on emotional empathy.

Despite these limitations our findings assist us in understanding the relationship between the IFG and empathy and offer evidence of the potential contributions of HD-tDCS in the realm of social cognition. As far as we know this is the first study to explore the role of the right IFG in empathy via HD-tDCS. Future studies may use larger samples or different empathy tasks to validate our findings. In addition, many studies suggest that the left IFG plays an important role in empathy (Lawrence et al., 2006; Jabbi et al., 2007; Hooker et al., 2008, 2010; Greimel et al., 2010; Sassa et al., 2012) and future research can explore the modulatory effects of HD-tDCS on empathy by targeting the left IFG to further examine the relationship between the IFG and empathy. Finally, studies have shown that many psychiatric patients and particularly patients with schizophrenia suffer from a serious empathy disorder and that cognitive empathy is more severely impaired in psychiatric patients (Dapretto et al., 2006; Derntl et al., 2009; Smith et al., 2015). Future studies can elaborate upon our research by investigating empathy in such patients and the role of anodal stimulation while further exploring the clinical significance of HD-tDCS in improving social cognitive abilities.

## Conclusion

Our findings confirm that the cathodal HD-tDCS of the right IFC can lead to an impairment of cognitive empathy while anodal stimulation can spur an improvement. However, the active HD-tDCS of the right IFC has no effect on emotional empathy. In summary, we believe that the IFG plays an important role in cognitive empathy and that HD-tDCS can be effective in modulating social cognitive abilities.

## Author Contributions

The experiment was designed by XW, FX, CX and KWang. WH, KWan, XC, SX and GX performed data collection and analysis. XW wrote the article. LW, G-JJ, FY and CZ were responsible for modifying the article. All of the authors made their own contributions to the final article and all have agreed to the submission of this version.

## Conflict of Interest Statement

The authors declare that the research was conducted in the absence of any commercial or financial relationships that could be construed as a potential conflict of interest.
